# Integrated Forming and Surface Engineering of Disc Springs by Inducing Residual Stresses by Incremental Sheet Forming

**DOI:** 10.3390/ma12101646

**Published:** 2019-05-20

**Authors:** Ramin Hajavifard, Fawad Maqbool, Anke Schmiedt-Kalenborn, Johannes Buhl, Markus Bambach, Frank Walther

**Affiliations:** 1 Department of Materials Test Engineering (WPT), TU Dortmund University, Baroper Str. 303, D-44227 Dortmund, Germany; anke.schmiedt@tu-dortmund.de (A.S.-K.); frank.walther@tu-dortmund.de (F.W.); 2 Chair of Mechanical Design and Manufacturing, Brandenburg University of Technology Cottbus-Senftenberg, Konrad-Wachsmann-Allee 17, D-03046 Cottbus, Germany; fawad.maqbool@b-tu.de (F.M.); johannes.buhl@b-tu.de (J.B.); bambach@b-tu.de (M.B.)

**Keywords:** disc spring, residual stress adjustment, incremental sheet forming, metastable austenitic stainless steel (MASS), martensite transformation, numerical modelling

## Abstract

Disc springs are conical annular discs, which are characterized by a high spring force with a small spring travel and good space utilization. In operation, they must meet high demands on the stability of the spring characteristic and the fatigue strength. Under loading, tensile stresses occur which limit the possible applications of disc springs. Compressive stresses can be generated in the stressed areas by means of shot-peening in order to extend the operating limits for a given yield and fatigue strength. Since the spring geometry and characteristics change during shot-peening, the design of the shot-peening treatment is iterative and cumbersome. The present research proposes an incremental forming process for forming and integrated targeted adjustment of residual stresses in disc springs from metastable austenitic stainless steel (MASS), to achieve improved spring properties and high cyclic strength. The main mechanism of residual stress generation is the transformation of metastable austenite into martensite under the action of the forming tool. Different experimental characterization techniques like the hole drilling method, X-ray diffraction, disc compression tests, optical microscopy and cyclic tests are used to correlate the residual stresses and disc spring properties. A numerical model is developed for simulating the martensite transformation in disc springs manufacturing. The results prove that incremental forming enables process-integrated engineering of the desired compressive residual stresses, entailing a higher spring force of metastable austenitic disc springs in comparison to conventional disc springs. Due to martensite formation, the generated residual stresses are stable under cyclic loading, which is not the case for conventionally manufactured springs.

## 1. Introduction

Disc springs are conical discs, finding application in cases which require high spring forces in limited installation space. In turn, disc springs are expected to have a high fatigue strength with low stress relaxation [1]. The fatigue life and stress relaxation behavior of a disc spring are determined by its residual stress state and the service loads. Tensile residual stresses are present in the tensile-loaded underside of conventionally formed disc-springs. They can cause early fatigue failure of the components under service loading [2]. Compressive residual stresses increase the fatigue life by lowering the mean stress [3,4]. In this regard, the fatigue life of a disc spring can be improved by using post forming shot-peening [5]. In shot-peening, small metallic balls impact the component surface at high velocity, causing plastic straining, near-surface compressive residual stresses, and the retardation of fatigue crack growth [6].

The most common choice of materials for the manufacturing of the disc springs are metastable austenitic stainless steels (MASS). MASS undergo strain-induced martensite transformation under plastic deformation, occurring either during the forming process or under service loads. Due to this, MASS are classified as transformation induced plasticity (TRIP) steels. The TRIP effect causes an excellent combination of strength, ductility and resistance to stress relaxation [7]. In MASS, austenite is stable at higher temperatures, whereas martensite is stable at lower temperatures [8]. In the absence of external forces, the critical free energy difference to start the phase transformation is attained at the martensite start temperature, $M_{s}$. The martensite transformation can be supported by mechanical driving forces, which can be provided as plastic work during forming operations or during service loading [9]. In MASS, the austenitic γ-phase is the parent face-centered cubic (fcc) phase. Two types of martensite transformations occur in MASS, i.e., γ to ε and γ to $\alpha^{\prime }.$ ε-martensite has a hexagonal closed packed (HCP) crystallographic structure, whereas the $\alpha^{\prime }$-martensite has a body-centered tetragonal (BCT) structure. The $\alpha^{\prime }$-martensite is of particular significance, as it increases the work hardening. This increase in work hardening increases resistance to mechanical instabilities and necking, causing an increase of ductility and strength [10].

The combination of MASS and shot-peening is used to increase the fatigue life of a component by inducing compressive residual stresses by deformation induced martensite transformation (DIMT). However, only a few publications deal with the shot-peening of MASS [11,12,13,14]. The main conclusion that can be derived from these studies is that the shot-peening of MASS creates a near-surface layer with a thickness of 20–40 µm, with mechanical properties different from the bulk material. A high magnitude of martensite and compressive residual stresses are found in this layer. The highest compressive residual stresses are found in this layer at the location where the martensite content is highest. This transformation has a positive effect on fatigue properties. However, no practical application of the shot-peened MASS components under static or dynamic loading, nor the behavior of the residual stresses have been reported, i.e., whether the residual stresses are stable under different loading conditions or not.

Furthermore, there are certain disadvantages associated with the shot-peening process. Due to the stochastic nature of the process, the resulting mechanical properties of the components are highly dependent on the selected process parameters [11]. If process parameters are not selected properly, it can deteriorate surface roughness and can cause a decrease in fatigue life [15,16]. With regards to disc springs, Doman et al. [17] reported an increase in the force-displacement curve for the shot-peened diaphragm air spring. However, a significant change in the free height of the disc spring was noted. Furthermore, the residual stresses generated by shot-peening disappeared under cyclic loading. Another major disadvantage of shot-peening is that the distribution of the residual stresses cannot be controlled. The radial and tangential residual stress components created by shot-peening are of the same magnitude. The residual stresses in the radial direction can significantly change the spring height. As an optimal condition, the residual stresses generated by shot-peening in the radial direction should be minimal. This control, however, is not possible with shot-peening.

In the current study, the incremental sheet forming (ISF) process is put forward as an alternative forming and surface engineering approach to manufacture and improve the mechanical properties of disc springs. In ISF, a small hemispherical tool locally deforms a sheet blank along a prescribed path, which is the contour of the final formed part. The localized forming mechanism allows higher formability but at the same time, it results in a high magnitude of the residual stresses [18]. These residual stresses are a significant factor for increased geometric deviations of the incrementally formed parts [19]. Different studies investigate the development of these residual stresses in ISF [20,21]. Furthermore, strategies like stress-relief annealing are reported to relieve the intensity of the residual stresses and increase the geometrical accuracy [22,23]. However, no study dealing with a targeted adjustment of the residual stresses and the development of the residual stresses in MASS by ISF has been reported. The only study identified in the literature dealing with ISF and MASS was proposed by Katajarinne et al. [24]. They developed a novel approach for regulating the mechanical properties, i.e., strength and ductility during the incremental forming of MASS by controlling the formation of strain-induced martensite.

No study deals with the development of residual stresses in MASS during ISF. Similarly, the behavior of the near-surface compressive residual stresses generated by shot-peening under static and cyclic loads has not been characterized. 

In view of these knowledge gaps, the first goal of the present study is to achieve the simultaneous integrated forming as well as surface engineering of disc springs. In this regard, ISF is used to form and induce the residual stresses in the disc springs. Here, the question is which process set-up and which process parameters should be used to form the springs and create the desired residual stresses. The mechanical properties of the formed disc springs are then characterized and compared to the conventionally formed disc springs. The second goal is to analyze the behavior of the residual stresses during cyclic loading, which in turn is dependent on the martensite stability, i.e., do the generated residual stresses along with deformation-induced martensite remain stable under cyclic loading or do they vanish with the increasing number of load cycles? The third goal of the current study is to develop a numerical model of the forming process of disc springs from MASS by ISF. The numerical model shall help predict and optimize the spring properties.

## 2. Materials and Methods 

### 2.1. Material and Geometry

**Figure 1:**
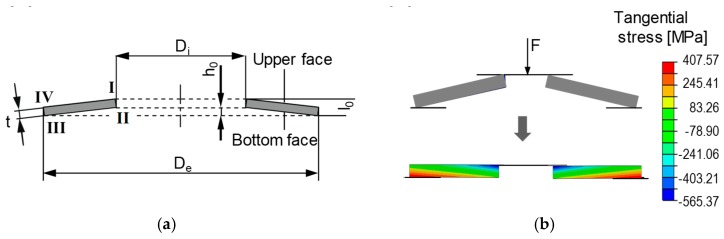
(**a**) The schematic of a conventional disc spring geometry. (**b**) Development of the stresses in disc springs under normal loading condition.

The incrementally formed disc springs were manufactured from the sheet blanks of two austenitic stainless steels AISI 301 (EN.1.4310) and AISI 316 (EN. 1.4401). These sheet blanks were produced by a commercial steel manufacturer Stahlbecker GmbH (Heusenstamm, Germany). The sheet blanks of thickness 1 mm and 3 mm were purchased for AISI 301 and AISI 316 steel respectively. The AISI 301 is designated as steel A and AISI 316 as steel B throughout the text. The chemical composition of both steels is presented in Table 1. The geometries to be manufactured are selected according to DIN 2093 [25]. The schematic of a typical disc spring is shown in Figure 1. The terms D_e_, D_i_, t, and l_0_ represent the external diameter, internal diameter, thickness and free height of the disc spring, respectively. The 2D stress state of a disc spring under normal loading conditions is presented in the Figure 1a. The bottom face of the disc spring is under circumferential tension and the upper face under compression (Figure 1b). 

For steel A, the selected geometry to be manufactured by ISF is 80/36/3 mm and 80/36/1 mm (D_e_/D_i_/t), whereas for steel B, the selected geometry is 112/57/1 mm (D_e_/D_i_/t). The conventionally formed disc springs in selected geometries from steel A and B were purchased from a commercial disc spring manufacturer. The conventionally formed disc springs are used for comparing the mechanical properties with incrementally formed disc springs. Furthermore, the reason for using various geometries with different thicknesses is that two different residual stress measurement techniques, i.e., hole drilling method and X-ray diffraction (XRD) are used in the current study. For XRD, the thickness of the sample does not influence the measured residual values. However, the hole drilling method is recommended for measuring residual stresses in the samples having a thickness greater than 2.5 mm. In addition, the reason for using two materials is to differentiate the role of martensite transformation and plastic deformation on the residual stresses. For AISI 301, the residual stresses are due to martensite transformation and plastic deformation and for AISI 316, the residual stresses are mainly due to plastic deformation. Hence, the role of martensite transformation on the residual stresses can be studied by comparing the results of these two materials. Disc springs are manufactured in each geometry and material. Afterwards, residual stress measurements in 3 mm thick disc springs and 1 mm thick disc springs are carried out by the hole drilling method and X-ray diffraction, respectively. These springs are then subjected to quasi-static and fatigue testing. The numerical framework is developed for steel A. An overview of the characterization techniques carried out for the disc springs manufactured from each material is presented in Table 2.

### 2.2. Integrated Forming with ISF

In ISF, a small hemispherical tool locally deforms the sheet blanks along a prescribed path, which is the final contour of the part to be formed. There are two main variants of the ISF process, single point incremental forming (SPIF) and two-point incremental forming (TPIF). Previous work on disc spring manufacturing revealed that the TPIF variant is more suitable [26]. 

**Figure 2:**
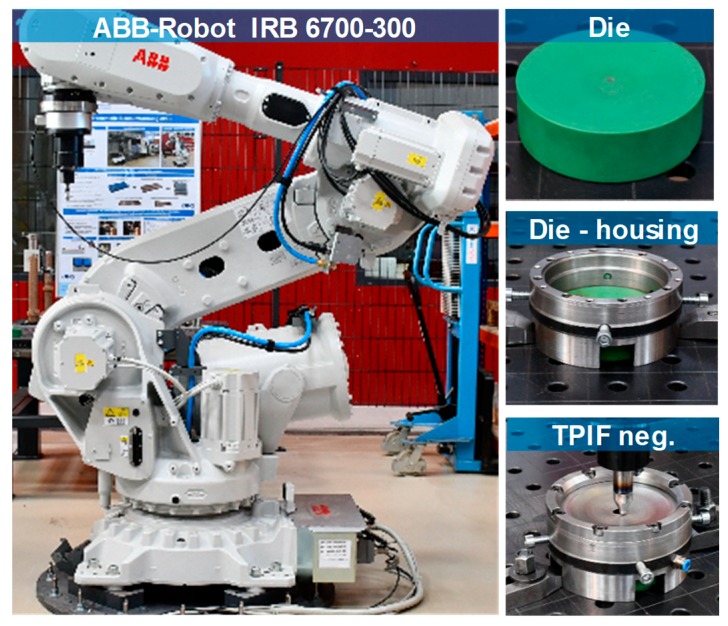
Experimental set-up of two-point incremental forming (TPIF) with a negative die using ABB-Robotic arm.

The experimental set-up of the TPIF process with a negative die is presented in Figure 2. The forming of the sheet blanks of steel A and steel B into incrementally formed disc springs is carried out using an ABB industrial robot and a hemispherical forming tool. Modular tooling with exchangeable Sika^®^ block inserts is used as a negative die. The Sika^®^ die force fits into a steel housing. The sheet blank is then clamped between a steel ring and housing containing the die. The clamped sheet blank is formed into the disc spring by the plastic deformation induced by the hemispherical tool. Furthermore, the die, housing, and the steel ring are manufactured for each spring geometry. The disc springs from steel A and steel B are then manufactured under the variation of tool diameter (5, 7.5, 10 mm) and tool step-down (0.05, 0.1, 0.2 mm). The central hole is trimmed after incremental forming to obtain the final shape of the disc springs. 

### 2.3. Residual Stress Measurements

**Figure 3:**
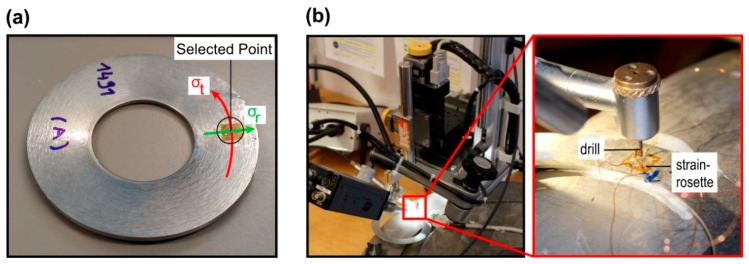
(**a**) Selected point for the measurement of the residual stresses, and (**b**) experimental set-up of the hole drilling method.

Hole drilling method. This method is based on the step by step drilling of a small hole and recording the respective relieved strains by use of a strain-gauge rosette. The measured strains are then converted into stresses. The residual stress analyzer from the company MTU^®^ was used to analyze the residual stresses at a specified point for all the disc springs of 3 mm thickness from steel A (Figure 2). The drilling in the specimen was carried out at room temperature and at 200,000 rpm to avoid the creation of the supplementary stresses. The diameter of the drill head was 0.8 mm. The radial and tangential components of the residual stresses $\sigma_{r}$ and $\sigma_{t}$ respectively were obtained from the measured strain by using the following formulation where E is the elastic modulus, having a value of 210 GPa and *A*, *B* are constants having values of −0.225 and −0.18803 respectively. The values of these constants are provided by the manufacturer of the residual stress analyzer system. Further, $\varepsilon_{r}\;,\;\varepsilon_{t}$ and $\varepsilon_{rt}$ are the in-plane strains, where $\varepsilon_{r}$ is the strain along the radial direction, $\varepsilon_{t}$ is the strain along the tangential direction and $\varepsilon_{rt}$ is the strain at 45° to the tool motion direction, $\sigma_{r}$ and $\sigma_{t}$ are the in-plane stresses corresponding to $\varepsilon_{r}$ and $\varepsilon_{t}$, respectively. The out-of-plane stresses along the thickness direction cannot be measured by this method and are hence neglected. The experimental set-up of the hole-drilling-method for measuring residual stresses in the disc springs is presented in the Figure 3.

(1) $$\begin{array}{c} \sigma_{r}\\ \sigma_{t} \end{array}=\;E\{\frac{\varepsilon_{r}+\;\varepsilon_{t}}{4A}\;\begin{array}{c} +\\ - \end{array}\;\frac{1}{4B}\sqrt{{\left(\varepsilon_{r}-\varepsilon_{t}\right)}^{2}\;+\;{\left[2\varepsilon_{rt}-\left(\varepsilon_{r}+\varepsilon_{t}\right)\right]}^{2}}\;\}$$

**Figure 4:**
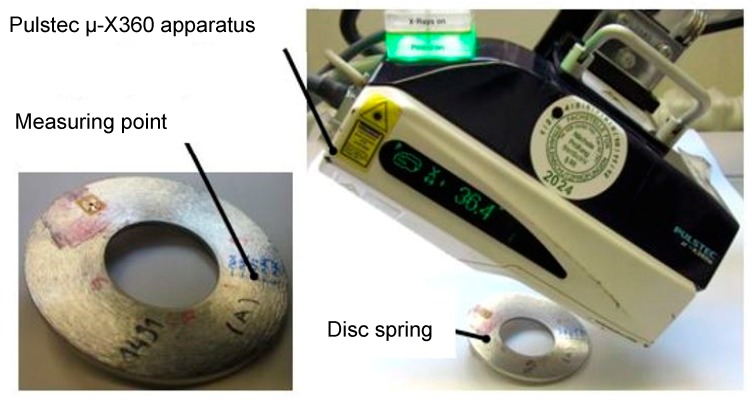
Experimental setup for the measurement of the residual stresses in disc springs by portable residual stress analyzer.

X-ray diffraction (XRD) Method. This method is a non-destructive residual stress measurement technique that is based on the measurement of the respective distance between the crystallographic planes. The spacing between the crystallographic planes is used as a measure of strain. The magnitude of the residual stress is measured by the comparison of the lattice plane spacing between the stressed and stress-free state of the material. The magnitudes of the residual stresses in disc springs manufactured from steel A with a dimension of 80/36/1 mm and steel B with a dimension of 112/57/1 mm were measured by using this technique. For this purpose, a portable X-ray residual stress analyzer μ-X360 from Pulstec industrial Co., Ltd. (Hamamatsu, Japan) was used. The stress analyzer is also capable of measuring the amount of the retained austenite in the MASS. The stress analyzer system uses Cr Kα-radiation and cosα-method for the residual stress measurements. An X-ray incidence angle of ψ0 = 30° was chosen. For the retained austenite content, an incidence angle of ψ0 = 18° was used. The experimental set-up is presented in Figure 4.

### 2.4. Quasi-Static and Cyclic Testing

**Figure 5:**
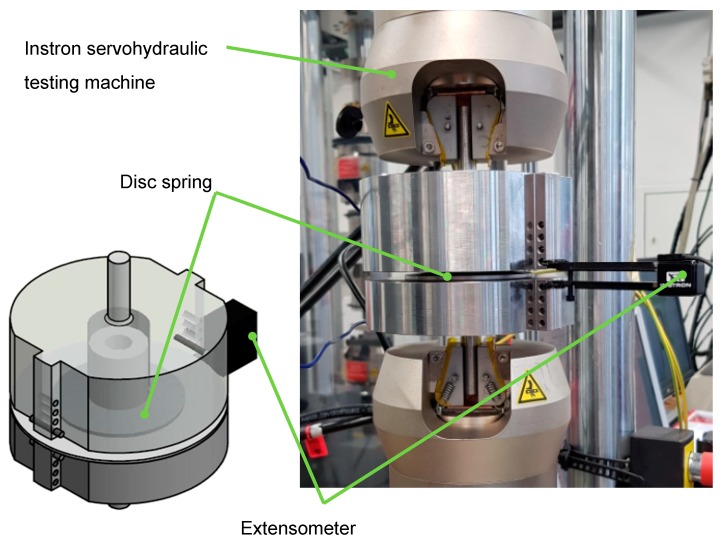
Experimental set-up for the quasi-static and cyclic testing of the disc springs.

The mechanical tests were carried out using a servo-hydraulic testing system (F = ±10 kN). To achieve clamping of the disc spring with appropriate boundary conditions, a fixture was manufactured and attached to the testing system. The experimental set-up is presented in Figure 5. The quasi-static tests on the conventional and incrementally formed disc springs under varying process parameters were performed by compressing the disc springs to a flat position and measuring the respective force-deflection curves. For this purpose, an extensometer with a gauge length of 12.5 mm and a strain rate of 0.0025 s^−1^ were used. In this way, the respective comparison of characteristic force-displacement curves of the conventional and incrementally formed disc springs as well as the influence of process parameters on the characteristic force-displacement curves were determined. 

The cyclic testing on the disc springs was performed using the same experimental set-up. The cyclic testing was carried out using the sinusoidal load time function with a load ratio of R = 10 and a frequency of 10 Hz. The maximum number of cycles for the fatigue testing of the conventional and incrementally formed disc springs was set at 2.5 × 10^6^ cycles. In addition, cyclic testing was performed until 100 cycles and was consecutively interrupted after every 1, 10 and 100 cycles to measure the residual stresses by using the XRD set-up presented in Section 2.3. This testing technique was applied to analyze the stability of the residual stresses in MASS under cyclic testing.

### 2.5. Microstructure and Material Characterization

The microstructure of initial sheet blank, incrementally formed disc spring after forming and after fatigue testing was investigated for both steels. For this purpose, micro-sections were cut radially and etched using Beraha-II etchant for 8 s. For microstructure analyses, a Zeiss Axio Imager light microscopy (Oberkochen, Germany) was used. The inspections were focused on the surface layer of the bottom side of springs, which was in contact with the forming tool. 

**Figure 6:**
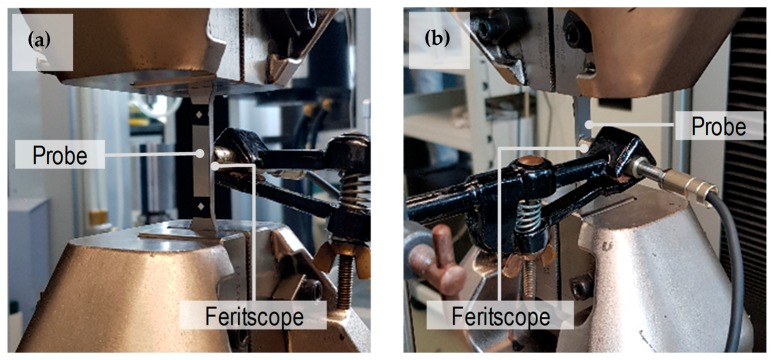
(**a**) Tensile test with a probe for in-site ferromagnetic content measurements (**b**) stress-strain response and evolution of martensite content as a function of plastic strain.

For material characterization, tensile test with in-situ ferromagnetic measurement was conducted for steel A to determine the evolution of the martensite volume fraction as a function of the plastic strains. The samples were taken from sheet blanks of steel A with a thickness of 3 mm and tensile tests were carried out by the universal testing machine Shimadzu AG-X (Kyoto, Japan) rated at 1200 kN. The ferromagnetic content was measured online with a Fisher Feritscope FMP-30. The martensite content was obtained from the ferromagnetic content by using the formulation proposed by Talonen et al. [27]. The experimental set-up is presented in Figure 6.

## 3. Material Model and Numerical Simulations

The material model is developed for the steel A in the current study. A macroscopic elasto-plastic model considering the phase transformations and the individual response of each phase is considered. The model takes into consideration the austenite phase of volume fraction $1\;-\;f_{\alpha^{\prime }}$ and the $\alpha^{\prime }$-martensite phase of volume fraction $f_{\alpha^{\prime }}$ which nucleates from the austenite phase. The transformation kinetics of the martensite is combined with a constitutive law and implemented as a material subroutine in the commercial FE-software LS-Dyna^®^. The model is implemented for volume elements. The mathematical and incremental formulation for martensite transformation and the constitutive law is presented in the following sections. Afterwards, the developed subroutine is used to determine the unknown material parameters followed by the numerical simulation of the disc springs.

### 3.1. Kinetics of Martensite Transformation

A sigmoidal function proposed by Olson and Cohen referred to as the OC-model was used to describe the martensite evolution as a function of plastic strains [28]. The model uses shear-band interaction as a dominant mechanism for the nucleation of the strain induced martensite. The relation between the volume fraction of the martensite and the plastic strain is described as [28]

(2) $$f_{\begin{array}{c} \alpha^{\prime } \end{array}}=1-exp\{-\beta [1-exp{\left(-\alpha \varepsilon \right)}^{n}\}$$

The parameter α controls the rate of shear-band formation with increasing strain and is dependent on the stacking fault energy. The parameter β is controlled by the chemical driving force of $\gamma \to \alpha^{\prime }$ transformation and its linear variation controls the probability of a martensite nucleus being generated by a shear-band interaction and n is a fitting parameter, whose value is taken as 2 [29]. The incremental formulation of the OC-mode implemented in the user-routine is presented:

(3) $$\dot{f_{sb}}=\;\alpha \left(1-f_{sb}\right)\dot{\varepsilon_{p}}$$

(4) $$A\;=\alpha \beta n{\left(f_{sb}\right)}^{n-1}\left(1-f_{sb}\right)$$

(5) $$\dot{f_{nm}}=\;A\left(1-f_{nm}\right)\dot{\varepsilon_{p}}$$

(6) $$V_{nm}\;=V_{a0}f_{nm}$$

where $f_{sb},\;f_{nm}$ represent the normalized shear band and transformed martensite volume fraction. $V_{a0},\;V_{nm}$ represent the initial austenite and new martensite volume fraction. The transformation kinetics also requires a plastic strain increment $\dot{\varepsilon_{p}}$, provided by the constitutive framework. 

### 3.2. Constitutive Law

The macroscopic flow stress of the steel A is obtained by the following relation:

(7) $$\sigma_{y}\;=\;\sigma_{y,\gamma }\left(1-f_{\alpha^{\prime }}\right)+\;\sigma_{y,\alpha^{\prime }}f_{\alpha^{\prime }}$$

where $\sigma_{f,\gamma }$ and $\sigma_{f,\alpha^{\prime }}$ are the flow stress of the austenite and martensite phase respectively. A von Mises yield function F is combined with an isotropic hardening law for each phase as follows:

(8) $$F=\;\sigma_{eq}-\left(\sigma_{0}+R\right)=\;\sqrt{\frac{3}{2}S_{ij}S_{ij}}-\;\sigma_{y}$$

where $\;\sigma_{eq},S_{ij},\sigma_{0}\;\mathrm{and}\;R$ are the equivalent von Mises stress, deviatoric stress, initial yield stress and isotropic hardening parameter respectively. A Young’s modulus of 188 MPa for austenite and 217 MPa for the martensite is assumed [30]. According to Equation (7), an important input required for the material model is the flow stress of the austenite and martensite phase. An analytical approach proposed by Bouqerel et al. [31] is utilized in the current study to determine the flow stress of each phase. The flow stress of the martensite phase can be calculated as:

(9) $$\sigma_{y,\alpha^{\prime }}=\sigma_{0,\alpha^{\prime }}+\alpha \mu M\sqrt{B}\sqrt{\frac{(1-\exp\left(-Mf\varepsilon \right)}{fL}}$$

where $\sigma_{0,\alpha^{\prime }}$ is the initial yield stress of the martensite phase determined according to the formulation proposed by Rodriguez et al. [32]. Further, the term α is a constant and M,  μ,  B, L,  f are the Taylor factor, shear modulus, Burger vectors, mean dislocation free path and recovery rate respectively.

The flow stress of the austenite phase is determined according to Kocks-Mecking theory, which states that the dislocation density evolution is due to competition between the rate of production and annihilation of the dislocations is utilized. This fact is mathematically represented as [31]:

(10) $$\frac{d\rho }{Md\varepsilon }=\frac{1}{\lambda }+\frac{k}{b}\sqrt{\rho }-f\rho$$

λ is assumed to be equal to the grain size of the austenite phase given by:

(11) $$\lambda =d_{\;\lambda }^{3}\;\sqrt{1-f_{\alpha^{\prime }}}$$

The macroscopic stress can be calculated from the shear stress as follows:

(12) σ=Mτ

The macroscopic stress can be divided as the interaction between the dislocations and lattice fraction:

(13) $$\sigma =\sigma_{0}+\alpha MGb\sqrt{\rho }$$

**Figure 7:**
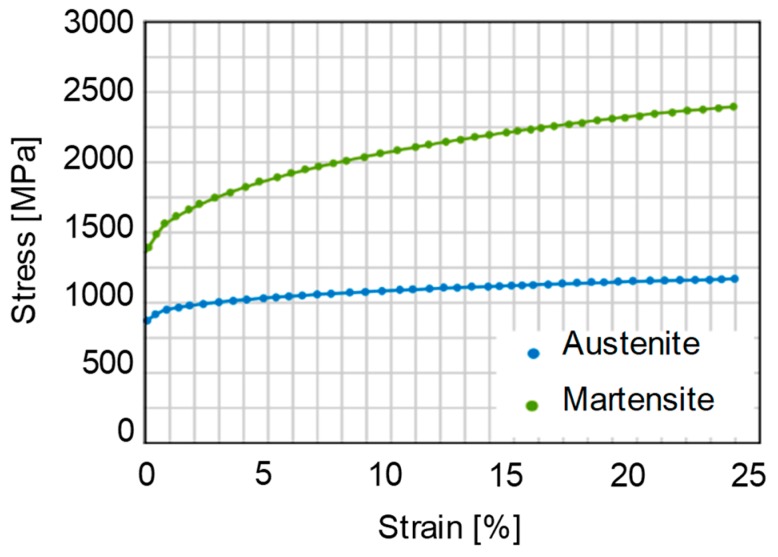
Flow stress curves of austenite and martensite phase.

Equations (10), (12) and (13) are used to determine the flow stress of the austenite phase. In the above equations, G is shear modulus, ρ is dislocation density, k and f are constants, M is the Taylor factor and α characterizes the dislocation-dislocation interaction. These parameter are taken from literature [31]. The calculated stress-strain curves for the martensite and austenite phase are presented in the Figure 7. 

### 3.3. Identification of Material Parameters

**Figure 8:**
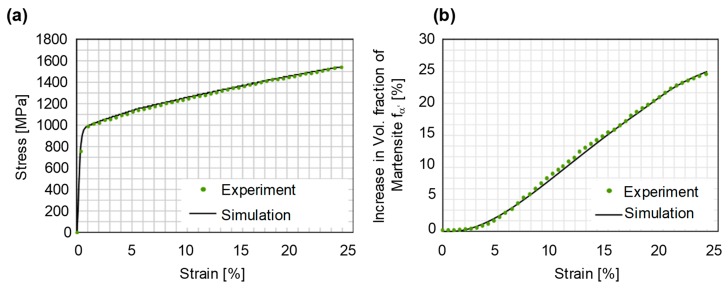
Comparison of experimental and optimized numerical curves: (**a**) Stress-strain, (**b**) evolution of the martensite content as a function of the plastic strain.

The three parameters (*α, β, n*) describing the evolution of the martensite content as a function of plastic strains were determined by using the experimental tensile test, feritscope data (Section 2.5) and an optimization procedure referred to as the inverse FE-approach. The inverse FE-approach is based on systematically varying the material parameters in the simulations until a good match is found between the numerical and the target experimental curves. The martensite transformation kinetics and the constitutive law is implemented as a vectorized user subroutine in the LS-Dyna^®^ software. For optimization, simulations are carried out on a single 8-node cubic element with full integration scheme. This helps to achieve the material parameters with a minimal computational cost. The optimized curves for the stress-strain response and martensite evolution are presented in Figure 8. A good match is found between both curves. The optimized values of the material parameters achieved for these curves are 4.25, 0.807 and 1.98 for *α*, *β* and n respectively.

### 3.4. Model Set-up for Disc Spring Simulations

**Figure 9:**
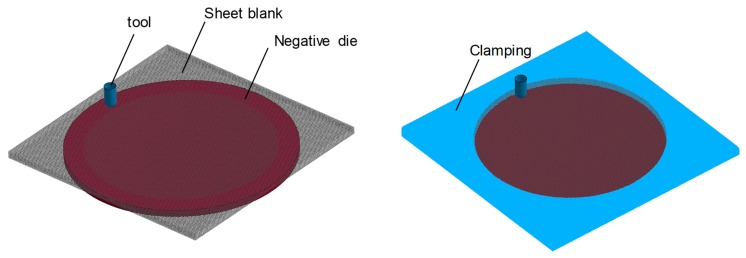
Model set-up for the disc spring manufacturing simulation.

The implemented material model as a user subroutine along with the identified material parameters was used to perform the numerical simulations of the disc spring manufacturing. The numerical model was so set-up that it replicates the experimental conditions. A rigid die is modeled to have a TPIF with a negative die set-up. The hemispherical tool is modeled as a rigid body. The volume elements are used for the numerical simulations. The sheet blank was modelled as a 110 × 110 mm plate. Modeling the sheet as a square plate helps obtain a uniform meshing size. For meshing, a 3D 8-node element with an edge size of 0.6 mm was used. The clamping of the sheet blank was set-up in such a way that it replicates the experimental conditions. Furthermore, an explicit time integration with a time step of 1e-06 s was used. The experimental tool path trajectories were converted into respective velocity curves and used as input for the tool motion in the numerical model [33]. The set-up of the numerical model is presented in Figure 9.

## 4. Results

### 4.1. Residual Stress Measurement

**Figure 10:**
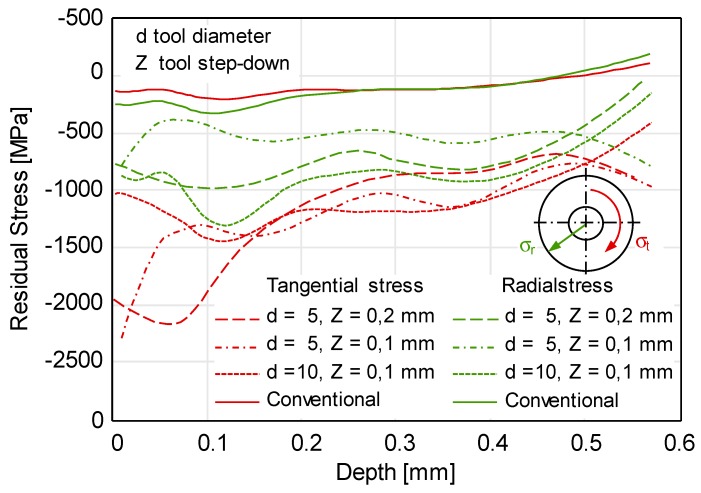
Residual stresses measured by the hole drilling method in conventional and in incrementally formed disc springs from steel A under varying process parameters.

The disc springs were manufactured from both steel A and B by varying the tool diameter and tool step-down and by using the TPIF with a negative die variant. After manufacturing, the residual stresses in disc springs from steel A and dimension 80/36/3 mm were measured at the center of the bottom face by using the hole drilling method. The measured values of the residual stresses are decomposed into tangential and radial components and plotted for varying process parameters in Figure 10. The distribution of the residual stresses indicates that the magnitude of the near-surface compressive residual stresses is high as compared to the conventional disc springs. The compressive residual stresses increase by increasing the tool diameter, whereas no significant difference in the magnitude of the residual stresses is recorded for varying tool step-down. Furthermore, an important observation from Figure 10 is that the magnitude of the tangential stresses is higher than the radial stresses, which in turn should have a positive effect on the spring properties. This is because the radial stresses can significantly distort the spring geometry. With conventional shot-peening, the magnitude of the radial and tangential component is the same. Hence, an important conclusion from Figure 10 is that forming with ISF allows controlling the magnitudes of the residual stress components. 

**Figure 11:**
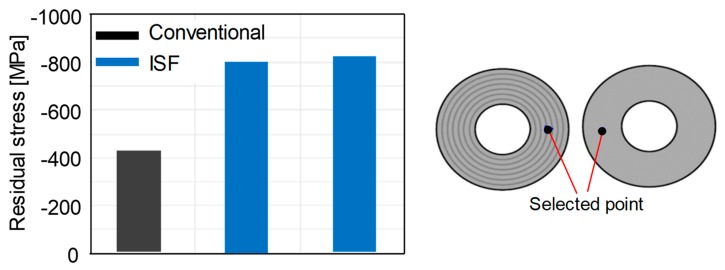
Repeatability of the residual stresses measured by X-ray diffraction (XRD) in conventional and incrementally formed disc springs from steel B under the same set of process parameters.

Furthermore, the residual stresses were measured using XRD technique in the conventional and incrementally formed disc springs from steel B and dimension 112/57/1 mm. The aim of these measurements was to check the repeatability of the residual stresses under a given set of process parameters. In this regard, the measurements were conducted on the center of the bottom face for a conventional disc spring and two incrementally formed disc springs manufactured under the same set of process parameters. The results are presented in Figure 11. It can be concluded that the residual stresses generated by the ISF process are repeatable under the same set of process parameters. Furthermore, similar to steel A, the magnitude of the compressive residual stresses on the bottom face is higher for incrementally formed disc springs for steel B in comparison to conventional disc springs.

### 4.2. Quasi-Static and Cyclic Response

**Figure 12:**
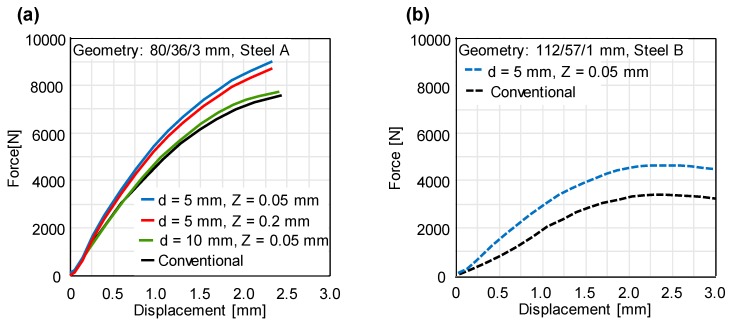
Characteristic force-displacement curves for conventional and incrementally formed disc springs from (**a**) steel A under varying process parameters (**b**) steel B.

Using the experimental set-up, the quasi-static disc compression tests are performed for conventional and incrementally formed disc springs under the variation of the process parameters. The characteristic force-deflection curves for steel A and B are presented in Figure 12. A significant increase in the spring force as compared to the conventional disc springs is achieved for both materials. Based on the results of residual stress measurement (Figure 10) and force-deflection curves (Figure 12), it can be stated that the higher the magnitude of the near-surface compressive residual stresses is, the higher the spring force. The highest increase in the spring force is obtained for the smallest tool diameter, which in turn has the highest near-surface compressive residual stresses. Although the magnitude of the residual stresses is the same for different tool step-downs, the smallest tool step-down has the highest spring force.

**Figure 13:**
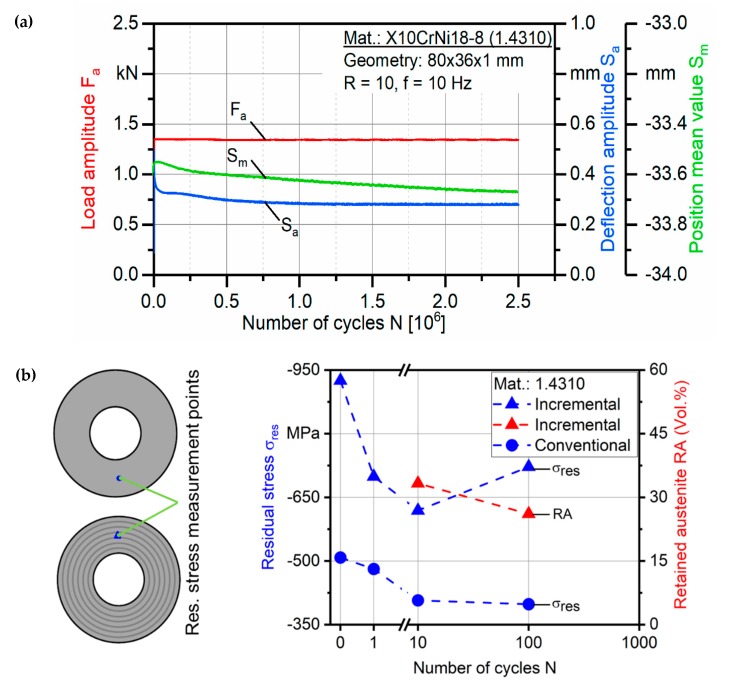
(**a**) Fatigue life investigation with a constant load amplitude for an incrementally formed disc spring of steel A. (**b**) Stability of the residual stresses in the first 100 loading cycles for steel A.

Fatigue testing using a constant load amplitude was carried out on the conventional and incrementally formed disc springs from steel A and B. The maximum number of cycles was set at 2.5 × 10^6^ cycles. No cracks or damage was observed for the conventional and incrementally formed disc springs from steel A and steel B after the end of maximum cycles. Figure 13a presents the load amplitude, resulted mean deflection and position values for incrementally formed disc springs from steel A. The minor fluctuations in the mean values of deflection and position, recorded at the beginning of the fatigue testing, were stablized afterward to provide a stable fatigue response over the whole range of cycles. The same effect was observed for steel B. Similarly, no failure was observed for the conventional disc springs. Hence, it can be stated as a conclusion that the fatigue life of the conventional and incrementally formed disc springs is comparable. 

However, a significant drop in the spring force and magnitude of the residual stresses occurs during the first loading cycles of the conventional disc springs [34,35]. This phenomenon is referred to as stress relaxation, which normally occurs due to local yielding and plastification of the small cross-sectional areas in the disc springs. In this regard, interrupted cyclic tests were performed to analyze the stability of the residual stresses and effect of deformation induced martensite transformation (DIMT) in stabilizing the residual stresses. Using XRD set-up, the magnitude of the residual stresses and retained austenite were measured after 1, 10 and 100 cycles in conventional and incrementally formed disc springs from steel A. The measurements were carried out on a selected point marked in Figure 13b. The results in Figure 13b indicate a drop in the magnitude of the residual stresses during the first 10 cycles for both disc springs. However, the martensite transformation indicated by a decrease in the volume fraction of retained austenite stabilizes the residual stresses for incrementally formed disc springs between 10 and 100 cycles. This is not observed for the conventionally formed disc springs. Hence, the residual stresses generated by ISF are stable due to martensite transformation and do not vanish under the cyclic loading for disc springs manufactured with ISF.

### 4.3. Evolution of the Microstructure

**Figure 14:**
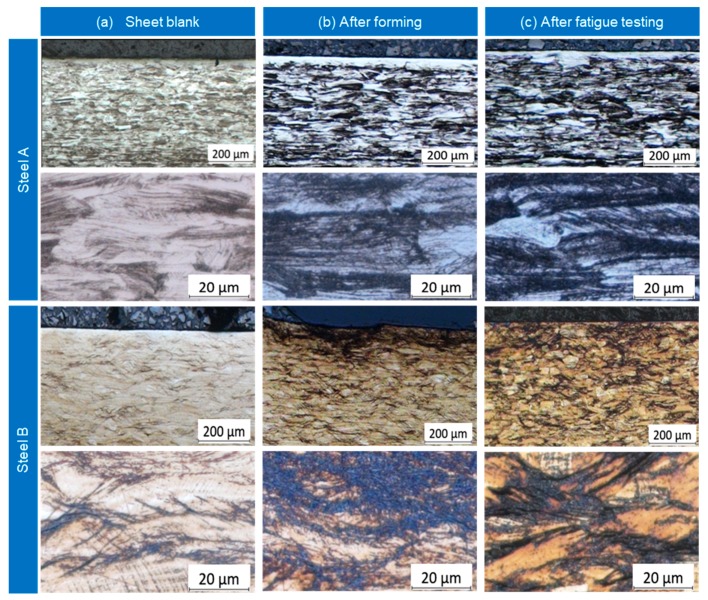
Micro-graphs of steel A and steel B cross-sections: (**a**) Sheet blank, (**b**) after forming, (**c**) after fatigue testing.

The microstructure of steel A and B was analyzed in the raw material, after forming and after fatigue testing for incrementally formed disc springs. The micrographs for all three stages for both steels are presented in Figure 14. The initial microstructure of steel A shows scattered martensitic islands, whereas a completely austenitic structure is observed for the steel B. A significant DIMT can be observed after the cyclic testing for both steels. However, for each steel, a different pattern is observed during the testing. The phase transformation in steel A takes place in the entire bulk of the disc spring and increases with increasing the number of cycles. For steel B the phase transformation starts at the tool-sheet contact surface and spreads towards the outer surface with an increasing number of cycles.

### 4.4. FEM Model Validation and Spring Properties

**Figure 15:**
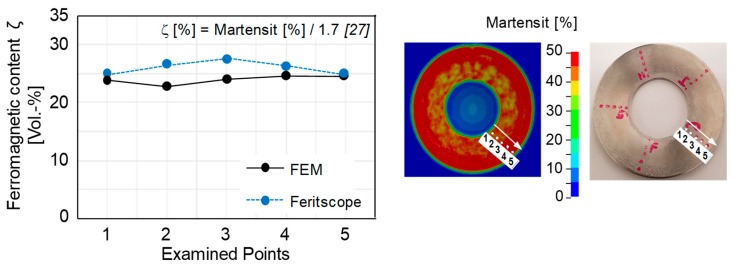
Comparison of the experimental and numerical ferromagnetic content measured at five locations on the bottom surface of the disc spring.

To analyze the spring properties from numerical simulations, the first step was to validate the numerical model. In this regard, the ferromagnetic content was measured at five distinct points located on the bottom surface of the disc springs by Feritscope measurements. These points are presented in Figure 15. Similarly, the martensite content at the same locations in the numerical simulation is converted into ferromagnetic content by using a calibration factor of 1.7, as proposed in the literature [27]. The calibration factor is necessary when converting ferromagnetic content into martensite. This is to minimize the measurement error of the Feritscope introduced due to magnetic permeability and also because it is a function of strain. Furthermore, the process parameters were the same for experiments and numerical simulations. The respective comparison shows that the measured experimental and numerical values correlate well. Hence, the model can be efficiently utilized to model the TRIP effect in MASS and to study the spring properties.

**Figure 16:**
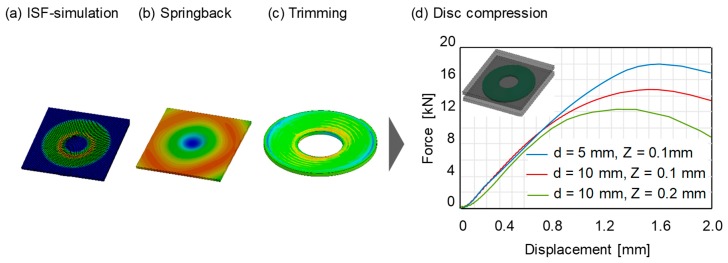
Numerical procedure for the disc compression test under varying process parameters.

Furthermore, the simulations were performed by varying the tool diameter and tool step-down. Afterward, disc compression simulations were performed to analyze the effect of each parameter on the spring force. This procedure was adopted to perform the compression tests as follows:

The springback simulation was performed after the disc spring manufacturing simulation. After the springback simulation, a trimming simulation was performed to obtain the final geometry of the disc spring. Finally, the trimmed disc spring was compressed between two rigid plates replicating the quasi-static compression test. The stress-strain histories for all elements were transferred between the springback, trimming and disc compression simulation. The procedure was repeated by varying the tool diameter and tool step-down in the simulations. The obtained spring forces under the influence of process parameters are presented in Figure 16. The obtained results are in qualitative agreement with the spring forces measured experimentally (Figure 12).

**Figure 17:**
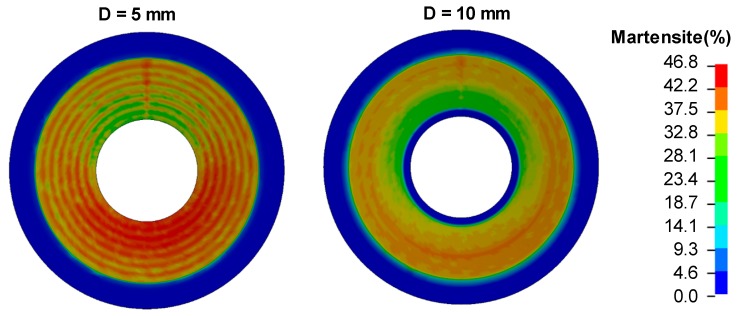
Comparison of the martensite content on the bottom surface of the disc springs for different tool diameters.

The spring force increases using a smaller tool diameter and tool step-down. Differences in the spring forces are due to the fact that the individual stress-strain response of each phase is separately considered in the user subroutine. Hence, each process parameter will create a different distribution of martensite and hence different spring properties. A comparison of the martensite content created by different tool diameters on the bottom face of the disc springs is presented in Figure 17.

## 5. Discussion

Disc springs were experimentally manufactured and simulated using a TPIF set-up with a negative die. The determination of the residual stresses and spring forces indicate enhanced mechanical properties for the disc springs manufactured by ISF as compared to the conventional manufacturing (forming + shot-peening). The reason for this observation is that the deformation induced by ISF results in dislocation multiplication and twinning on a global scale, whereas locally the hemispherical tool treats the disc spring surface similar to a deep rolling process. This, in turn, is possible by the presence of support in the form of a negative die. The tool then locally forms the sheet blanks and triggers the formation of deformation induced martensite in the tool-sheet contact zone. As the local forming mechanism is similar to deep rolling, the transformed martensite will be under compression. Hence, the DIMT in combination with global plastic straining leads to simultaneous integrated forming and surface engineering of the disc springs and produces high near-surface compressive residual stresses.

The conventional disc spring exhibits stress relaxation, i.e., a drop in the spring force over the first few cycles [34,35]. In the case of the incrementally formed disc springs, the cyclic testing indicates a stable response of the residual stresses. This is because the martensite does not vanish during cyclic testing, instead additional DIMT takes place, which stabilizes the spring behavior. The hemispherical tool moves by a sliding contact that flattens asperities but under repeated exposure could deteriorate the surface, and hence provide additional sites for fatigue crack nucleation and propagation. However, under the forming conditions used in this work, no fatigue failure of the incrementally formed disc springs was observed. 

A change in the tool diameter leads to a respective change in the size of the contact zone between the tool and the sheet blank. The smaller the tool diameter, the more concentrated the deformation, which leads to higher plastic strains. Higher plastic strains promote DIMT. Hence, a smaller tool diameter leads to higher spring force and vice versa. In the case of the different tool step-down, the DIMT will be the same in the contact zone but as the smaller tool step-downs results in a larger contact area, the global DIMT will be more for smaller tool step-downs. This, in turn, explains the different spring forces for different tool step-downs although, the measured residual stresses from the hole drilling method for varying tool step-down is nearly the same, due to the fact that the measurement reveals local properties in a small measurement volume. Hence, the spring force increases by increasing the tool step-down. Both experimental and numerical results of spring forces validate these observations.

However, in the current study, a qualitative validation of the experimental and numerical spring forces is achieved. A quantitative validation is not possible with the employed isotropic hardening model. This is due to the forming mechanism of ISF, which involves continuous cyclic bending and unending of the sheet metal [36]. The state of the residual stresses generated due to these cyclic effects cannot be accurately captured by an isotropic hardening model, but rather it requires a combined isotropic-kinematic hardening model. Furthermore, for an accurate description of residual stresses and spring forces, the cyclic model should also account for the martensite transformation effect in its formulation, particularly the coupling of back stress formulation and martensite fraction. The development of such a model is a point of focus for future research.

## 6. Conclusions

As an alternative to conventional forming and shot-peening, utilizing the ISF process allows for better control of spring properties and stability of residual stresses. Different characterization techniques were used to determine the static and cyclic properties of the disc springs. Furthermore, a numerical model considering the kinetics of martensite transformation is used to simulate the TRIP effect. The important conclusion from the current work are summarized as follows:

- The forming of disc springs with TPIF with a negative die results in higher compressive residual stresses and spring forces as compared to conventionally formed disc springs. Hence, the proposed methodology enables simultaneous integrated forming with surface engineering.
- The spring force increases with decreasing tool diameter and tool step-down for incrementally formed disc springs. Furthermore, based on the process parameters, the control of radial and tangential residual stresses can be achieved, which can help in maximizing the spring force or in designing the spring geometries with tight geometric tolerances.
- With regard to the cyclic testing, a stabilized fatigue and residual stress behavior of the incrementally formed disc springs is achieved. Although conventional and incrementally formed disc springs reach the same number of cycles (2.5 million) without failure, the residual stresses in conventionally formed disc springs vanish after a few cycles in contrast to the incrementally formed disc spring. The residual stresses do not vanish with an increasing number of cycles for incrementally formed disc springs and hence stabilize the force-displacement characteristics of the spring.
- A numerical model considering the kinetics of martensite transformation in combination with an isotropic hardening model is implemented in LS-Dyna. The model can accurately predict the martensite content as a function of plastic strain and is used to study the effect of process parameters on the martensite transformation and resulting spring properties. A good match is found with the experimental results.

## Figures and Tables

**Table 1:** Chemical composition of steel A and B (Alloying elements (wt.%)).

Steel	AISI	EN	C	Si	Mn	P	S	Cr	Ni	Mo	N	Fe
A	301	1.4310	0.07–0.15	1.0	2.0	0.045	0.03	17.5–19.5	6.5–8.0	-	0.1	Balance
B	316	1.4401	0.07	1.0	2.0	0.05	0.015	16.0–19.0	6.0–9.5	0.8	0.1	Balance

**Table 2:** Overview of Experimental and Numerical Method.

Material	Residual Stress Measurement		Quasi-Static Com-Pression Tests	Fatigue Test	Micro-structure	Tensile Test with Feritscope Measurements	FEM Sim.
	Hole Drilling Method	X-ray Diffraction					
Steel A	80/36/3	80/36/1	80/36/1	80/36/1	✓	✓	✓
Steel B	-	112/57/1	112/57/1	112/57/1	✓		
